# Personalized Disease Monitoring in Pediatric Onset Multiple Sclerosis Using the Saliva Free Light Chain Test

**DOI:** 10.3389/fimmu.2022.821499

**Published:** 2022-04-05

**Authors:** Esther Ganelin-Cohen, Evgeny Tartakovsky, Ely Klepfish, Sizilia Golderman, Ayal Rozenberg, Batia Kaplan

**Affiliations:** ^1^ Institute of Pediatric Neurology, Schneider Children’s Medical Center of Israel, Petach Tikva, Israel; ^2^ Sackler School of Medicine, Tel-Aviv University, Tel-Aviv, Israel; ^3^ Tartakovsky MLD Consultancy, Rishon Lezion, Israel; ^4^ Quant Aspect Limited, London, United Kingdom; ^5^ Heller Institute of Medical Research, Sheba Medical Center, Ramat Gan, Israel; ^6^ Department of Neurology, Rambam Health Care Campus, Haifa, Israel

**Keywords:** free light chains, multiple sclerosis, pediatrics, dimers, monomers, western blotting, Firth logistic regression

## Abstract

**Background:**

Development of new safe methods of monitoring disease activity in the pediatric onset multiple sclerosis (POMS) is a challenging task, especially when trying to refrain from frequent MRI usage. In our recent study, the *saliva* immunoglobulin free light chains (FLC) were suggested as biomarkers to discriminate between remission and active MS in adults.

**Objectives:**

To assess utility of saliva FLC measurements for monitoring disease activity in POMS.

**Methods:**

We used semiquantitative Western blot analysis to detect immunoreactive FLC monomers and dimers and to calculate the intensity of their bands. Statistical tests included Firth logistic regression analysis suitable for small sample sizes, and Spearman’s non-parametric correlation.

**Results:**

In *naive* POMS patients, the saliva levels of FLC in relapse were significantly higher than those in remission. Significant correlation was found between FLC levels (monomers, dimers or both) and the load of enhanced lesions in MRI scans. FLC levels may be reduced under treatment, especially as result of corticosteroids therapy. Follow-up of individual patients showed the correspondence of changes in the FLC levels to MRI findings.

**Conclusions:**

Our results show the potential of the non-invasive saliva FLC test, as a new tool for monitoring the disease activity in POMS.

## Introduction

Multiple sclerosis (MS) is a chronic not curable but manageable autoimmune inflammatory disease that causes damage to the myelin sheaths of nerve cells in the central nervous system. Typically, the onset of symptoms appears between the second and fourth decades of life (1). Pediatric onset MS (POMS) is an uncommon early manifestation of the disease. Case series in literature indicate that 5-10% of MS patients experience their first demyelinating event before age 18 (2–5). A recent systematic review and meta-analysis of the POMS epidemiology indicates the overall incidence ranged from 0.05 to 2.85 per 100,000 (6). In the recent years we have been facing an increasing incidence of POMS cases (7), which may be due either to growing awareness and accessibility of magnetic resonance imaging (MRI), or, possibly, as a consequence of environmental and other etiological factors. 

The diagnosis of MS is based on both clinical presentation of demyelinating events (which may be non-specific, but suggestive of MS), and dynamic MRI changes that meet the McDonald criteria (8–10). The McDonald criteria are based on demonstration of white matter lesions dissemination in time and space. In the latest 2017 version of the McDonald criteria (9), the presence of oligoclonal immunoglobulin bands (OCB) as a cerebrospinal fluid (CSF) marker of MS was added replacing the imaging parameter of “distribution in time”.

However, the diagnostic criteria are still evolving. For instance, myelin oligodendrocyte glycoprotein (MOG) antibody-associated disorders (MOGAD), which were an integral part of MS cases in the past, are now defined as a new CNS autoimmune entity. MOGAD, which is much more common in the pediatric population, can be confused with MS due to overlapping clinical presentation but dictates different usage of disease-modifying therapies (DMTs) (11).

POMS differs from adult MS in several aspects. Relapsing-remitting disease course is typical for POMS, although cases with primary progressive disease are reported recently (up to 7%) (12). POMS patients show more severe inflammation early in the disease course which is characterized by a higher rate of relapses and a higher load of lesions observed by MRI (13). Also, the long-term disability progression is relatively slow in children (14). Clinical manifestations vary from poly-symptomatic presentation, especially in the pre-pubertal children, to more monofocal appearance (such as optic neuritis and sensory symptoms) in the post-pubertal patients. In addition, posterior fossa, brainstem, and cerebellar involvement are more common in POMS than in adult patients (15).

MRI is mandatory for the initial diagnosis and helps to estimate the dynamic changes and aggravation of inflammation. At the beginning of the disease course, MRI is routinely used for monitoring the disease activity. Imaging can be utilized at reduced frequency in later stages with extended in-between intervals, if remission has been achieved. Many demyelinating MRI protocols require gadolinium-based contrast agents (16, 17). The frequent use of gadolinium raises safety concerns for the pediatric population - an increasing body of evidence suggests that gadolinium is deposited and retained in the brain (18, 19). Although the available data regarding the harm due to gadolinium exposure are inconclusive, there is a tendency to reduce such exposure to the absolute minimum. Given this limitation, it is necessary to find replacement means for monitoring disease activity. The laboratory CSF tests such as OCB or free light chain (FLC) analyses (20) require lumbar puncture, and therefore are impractical for disease monitoring. Moreover, it is not clear whether these diagnostic CSF markers of MS would be helpful for monitoring disease activity. Thus, development of safe and accessible alternative monitoring techniques remains to be challenging in the pediatric population. In our previous studies (21, 22) we found that analysis of saliva FLC is a promising non-invasive test to determine disease activity in adult MS patients. We showed that Western blot analysis of saliva FLC dimers and monomers was suitable for this purpose.

The present study is aimed at evaluating the utility of our saliva FLC test for monitoring the disease activity in POMS patients. Since our preliminary data showed that medications used to treat MS patients may affect the saliva FLC levels (22), we initially studied saliva FLC in *naive* pediatric MS patients. The data obtained for naive POMS patients were compared to those in pediatric patients experiencing other neurological diseases (both demyelinating and non-demyelinating). Application of Firth logistic regression (suitable for small sample sizes) for multivariate discrimination between relapse and remission states, followed by Spearman’s non-parametric test showed significant correlation of saliva FLC levels with clinical state (relapse or remission), as well as correlation with MRI findings. Having established this correlation, we analyzed changes in the FLC levels during *the follow-up of individual POMS patients* by matching these changes with clinical disease status, treatment and MRI findings. Based on the obtained results, we found that our saliva FLC test may serve as an easy, safe and non-invasive tool to monitor the disease activity in POMS.

## Materials and Methods

### Patients

This study involved 23 relapsing-remitting POMS patients (15 females), aged from 10 to 17 years (**Table 1**; **Supplementary 1**, **Tables S1** and **S2**). A group of patients with other demyelinating diseases (*n* = 28, aged from 10 to 17 years, 17 females, **Supplementary 1**, **Table S3**) included the following: clinically isolated syndrome (CIS, *n* = 18), radiologically isolated syndrome (RIS, *n* = 3), aquaporine-4-positive neuromyelitis optica (NMO/AQP4+, *n* = 1), acute disseminated encephalomyelitis (ADEM, *n* = 2) and myelin oligodendrocyte glycoprotein antibody disease (MOG+, *n* = 4). **Table 2** displays a group of 11 patients (8 females) with non-demyelinating neurological diseases (aged from 6 to 17 years). The study also included a control group of 14 healthy children (7-17 years old, 10 females), who showed no evidence of neurological, inflammatory, or any other chronic diseases.

**Table 1:** POMS patients: saliva FLC indices and clinical and radiological findings.

MS patient status	Samples number (*n*)		FLC indices		MRI findings				EDSS
			κ_D_ + λ_D_	κ_M_ + λ_M_	T2 load		Gd+ lesions		
					brain	spine	brain	spine	
**No treatment**									
**MS relapse**	14		1.05 – 19.66	1.14 – 22.13	2 - 29	0 - 6	0 - 4	0 - 2	0 -2
**MS remission**	10		0.65 – 4.52	0.39 – 4.52	1 -12	0-3	0	0	0
**Under treatment - DMT**									
**MS relapse**	4		0.16 – 12.06	0.51 – 12.31	7 - 20	0 - 5	0 - 3	0	1 - 2
**MS remission**	23		1 – 6.99	0 – 5.38	5 - >20	0 - 5	0 - 2	0	0 - 2

**Table 2:** Pediatric patients with non-demyelinating diseases: diagnosis, saliva FLC indices, and radiological findings.

Patient code	FLC indices						Diagnosis	Brain MRI:T2 lesions*
	κ_D_	κ_M_	λ_D_	λ_M_	κ_D_+λ_D_	κ_M_+λ_M_		
NDD 22	1.29	1.26	2.44	1.10	3.74	2.36	Rasmussen encephalitis	0**
NDD 42	1.13	0.25	0.88	0.00	2.02	0.25	Uveitis bilateral	0
NDD 26	1.29	1.26	2.18	1.10	3.48	2.36	Heterotropia	0***
NDD 33	1.19	2.31	1.72	1.34	2.91	3.65	DNET	7****
NDD 40	0.80	3.26	1.25	1.68	2.04	4.94	CADASIL	4
NDD 139	1.45	0.80	3.25	1.22	4.70	2.02	PANDAS	3
NDD 145	1.65	1.68	2.99	1.53	4.64	3.21	APLA	3
NDD 29	0.02	0.31	0.05	1.34	0.07	1.65	Sydenham chorea	9
NDD 149	2.35	0.86	1.72	1.34	4.06	2.20	IIH	0
NDD 150	1.31	0.17	0.96	0.80	2.28	0.97	Dystonia	0
NDD 151	2.01	3.76	2.39	1.74	4.40	5.50	Cavernoma	3

*not specific for demyelinating disorder; **cerebral hemiatrophy; ***gray matter findings typical for heterotropia; ****DNET left frontal + white matter lesions, DNET, Dysembryoplastic neuroepithelial tumors; CADASIL, Cerebral autosomal dominant arteriopathy with subcortical infarcts and leukoencephalopathy; PANDAS, Pediatric autoimmune neuropsychiatric disorders associated with streptococcal infections; APLA, Antiphospholipids antibodies syndrome; IIH, Idiopathic intracranial hypertension.

Saliva sampling was carried out between November 2016 and August 2020, during patient’s regular visits at the outpatient neurology clinic of a large tertiary medical center. Collection of samples was performed during the morning hours (from 8 to 12 a.m.) by spitting, and stored in 1.5 ml Eppendorf tubes at -30°C until use. In some cases, the saliva samples were taken repeatedly from the same patients during their follow-up period. Total number of tested saliva samples was *n* = 109, of which 54 were collected from POMS patients, 30 – from patients with other demyelinating diseases, 11 – from patients with non-demyelinating neurological diseases, 14 – from healthy children.

Diagnosis of MS was based on the revised 2017 McDonald criteria. A relapse was defined as new or worsening typical MS symptoms lasting for ≥ 24 h. On the day of saliva sampling, patients underwent clinical evaluation, and their expanded disability status scale scores (EDSS) and MRI data were documented.

### Sample Preparation and Western Blot Analysis

Saliva samples (approximately 0.5 ml) were collected and processed as described previously (22). Before testing, the saliva samples were centrifuged at 16 000 g for 20 min in the Eppendorf centrifuge (Eppendorf5415C; Marshal Scientific, Hampton, NH, USA). Samples were run under non-reducing conditions on 4 – 20% Nu-Sep Tris-Glycine gels (Gradipore, Frenchs Forest, New South Wales, Australia) and blotted onto a nitrocellulose membrane (Schleicher and Schuell, Dassel, Germany). FLC bands were detected using rabbit antibodies to human Ig κ and λ light chains (Dako, Carpinteria, CA, USA). Super-Signal West Pico Chemiluminescent Substrate (Pierce, Rockford, IL, USA) was used for band visualization.

### FLC Indices

Intensity *(I)* of the immunoreactive FLC bands was quantified by electrophoresis analysis software described previously (23). A reference sample, representing a mixture of the saliva samples from 10 healthy children, was included in each electrophoretic run alongside the tested samples. The quantified intensity values of FLC bands in the tested samples were normalized with respect to those of the reference sample: *I*_normalized_ = *I*_tested sample_/*I*_reference sample_. These normalized values were defined as FLC indices for the monomer and dimer levels (κ_M_, λ_M_, κ_D_, λ_D_). In addition, the sums of (κ_M_ + λ_M_) and (κ_D_ + λ_D_) were also calculated and used as total FLC monomer and dimer indices, M(T) and D(T), respectively.

### MRI Data

MRI scans were evaluated for the number of T2 lesions and *T1-gadolinium enhancing* lesions (Gd+) in the brain and spinal cord. To study the association between FLC levels and MRI findings in the relapse group we considered only the MRI scans obtained within six weeks from the relapse onset in accordance with the visualization timeframe of active MRI lesions (24).

### Statistical Analysis

Statistical analysis was performed using the JMP 15.1 software (SAS Institute Inc.) and R software (version 3.2.1; package *brglm* version 0.5-9). Firth’s penalized logistic regression analysis (25) suitable for small samples sizes was used to discriminate between the relapse, remission, and control groups. The Spearman’s non-parametric test was used to study the correlation between the saliva FLC data and the MRI findings.

## Results

### Saliva FLC Analysis Allows to Discriminate Between the Relapse and Remission States in POMS

**Figure 1:**
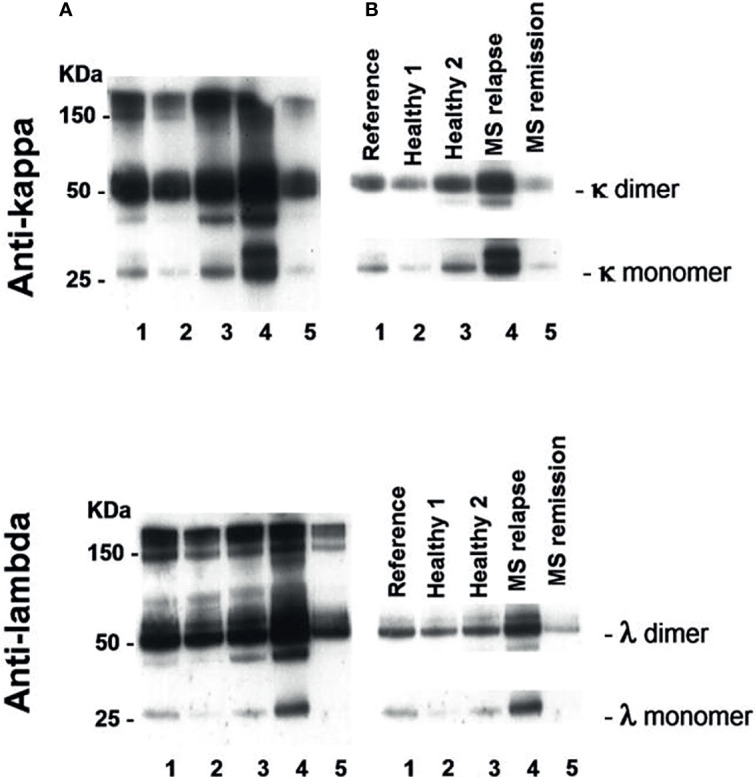
Western blot analysis of saliva FLC monomers and dimers in two healthy individuals (tracks 2 and 3), POMS patient in relapse (track 4) and POMS patient in remission (track 5). Track 1 - reference sample. **(A)** Exposure to X-ray film to view free and bound light chains: FLC monomers (25 kD), FLC dimers (50 kD), and intact immunoglobulins (≥ 150 kD). **(B)** Exposure to X-ray film to calculate intensity of FLC bands in a linearity range. The electrophoregram demonstrates high saliva FLC levels (monomers and dimers) in POMS patients in relapse as compared to those in the healthy individuals and in the POMS patients in remission.

An example of Western blot analysis of saliva FLC monomers (25 kDa) and dimers (50 kDa) is demonstrated in **Figure 1**. The levels of saliva κ and λ FLC monomers and dimers are high in non-treated patient in relapse (track 4) as compared to those in healthy individuals (tracks 2 and 3) and patient in remission (track 5).

Intensities of the FLC bands were measured in saliva of 14 healthy children (healthy group), and in saliva of 24 untreated MS patients of whom 14 were in relapse and 10 - in remission. Total FLC indices, namely: total dimer index D(T) **= (**κ_D_ + λ_D_) and total monomer index M(T) **=** (κ_M_ + λ_M_) were calculated for each individual. In the group of healthy individuals D(T) values varied from 0.05 to 3.53 (mean = 1.66; SD = 1.13), while in the MS relapse group D(T) values varied from 1.05 to 19.66. The M(T) values varied from 0.44 to 4.4 (mean = 1.89, SD = 1.19) in the healthy group, and from 1.14 to 22.13 - in the relapse group.

**Figure 2:**
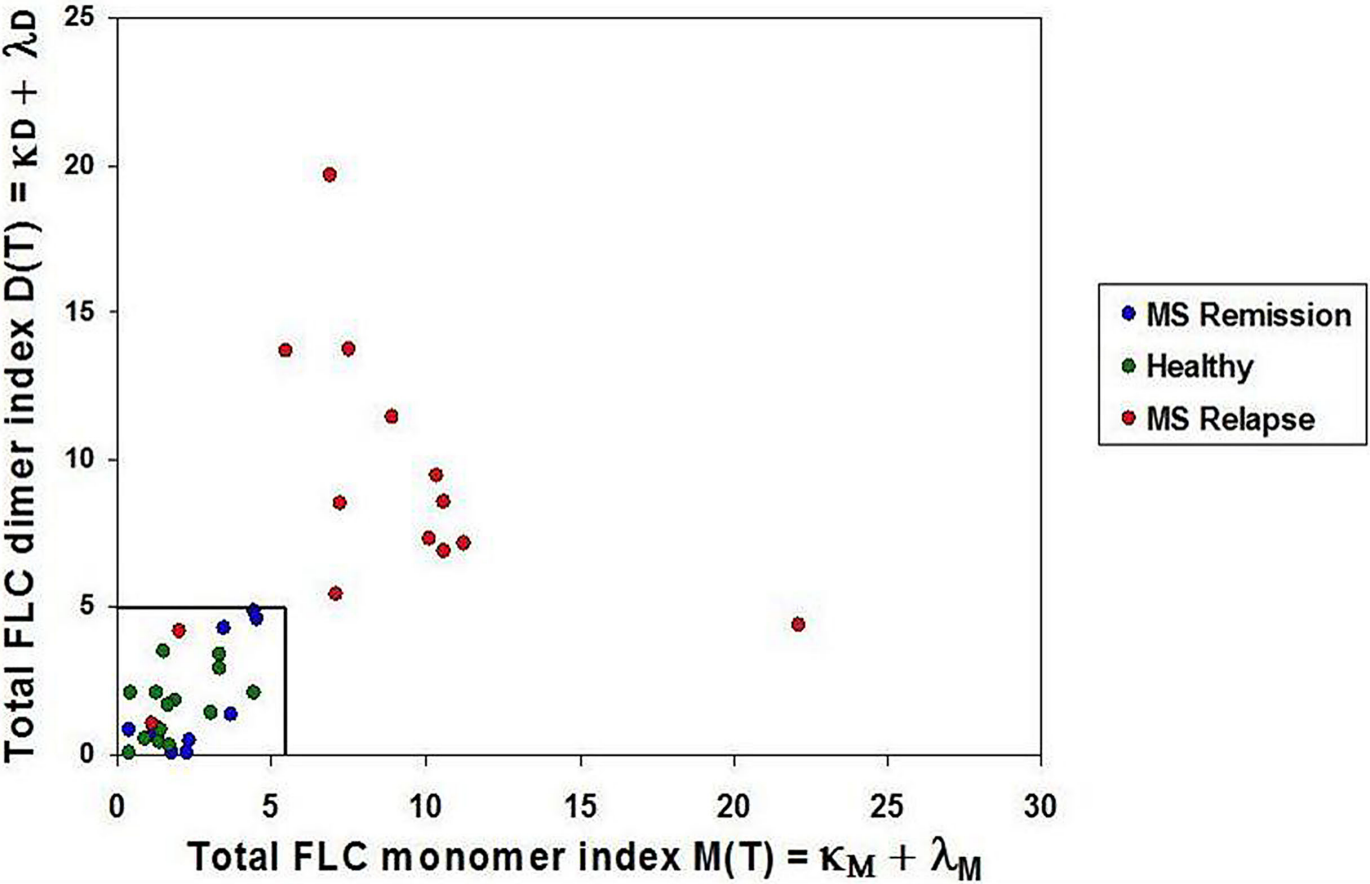
Saliva FLC indices in naive POMS patients. POMS patients in *relapse* - red circles; POMS patients in *remission* - blue circles; healthy children - green circles. Most of the POMS patients in *relapse* demonstrate the abnormally high values of the FLC indices. (D(T) > 5.0 and/or M(T) > 5.5) as compared to the healthy individuals and the POMS patients in remission.

Mean plus three SD values of FLC indices in the *healthy group* were used to define the upper limits of normal FLC index ranges: D(T) = 5.0 and M(T) = 5.5. Analysis of 14 MS relapse cases showed that in 12 of them FLC index values were above these limits: D(T) **>** 5.0 and/or M(T) > 5.5. In the MS remission group, the D(T) and M(T) values varied from 0.007 to 4.88 and from 0.39 to 4.5, respectively, thus being close to the values found in the healthy group (**Figure 2**, **Table 1** and **Supplementary 1, Table S1**).

The obtained data suggest the ability of the saliva FLC test to discriminate between relapse and remission states. This assumption was studied using a logistic regression model. This model made it possible to use a single variable/predictor *X* (the sum of M(T) and D(T)), for characterization of each case. The logit for this model was defined as a combined FLC-Firth index (cFLC-F); the coefficients of this model are equal to - 3.06 and 0.34 for the intercept (*β*_0_) and the slope (*β*_1_), respectively:


(Eq.1) cFLC–F=logit[Pr(Y=1)]=−3.06+0.34[D(T)+M(T)]

**Figure 3:**
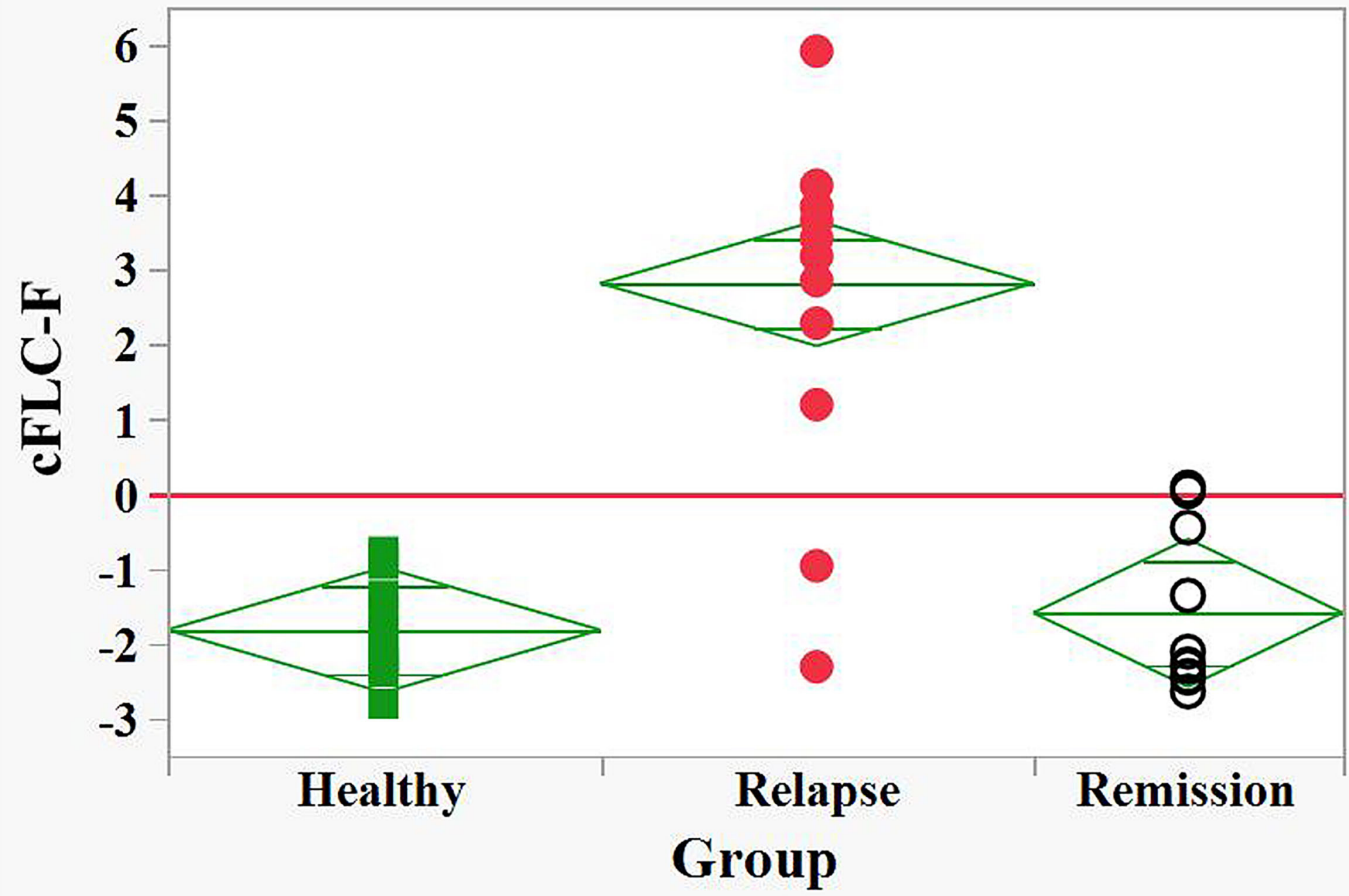
Discrimination between relapse and remision groups of naive POMS patients and healthy children by the FLC based Firth’s logistic regression function, cFLC-F. cFLC-F values were calculated for each individual (**Supplement 1**). “Relapse” - red filled circles, “Remission” - black empty circles, and “Healthy” groups - green filled squares.) Effective discrimination between healthy children and POMS patients in relapse, as well as between relapse and remission groups was observed (cut-off value, cFLC-F = 0).

cFLC-F values were calculated for each individual together with corresponding probabilities (**Supplementary 2** and **Table S2**). This allowed effective discrimination between the groups (**Figure 3**).

### Correlation Between cFLC-F Indices and MRI Findings

**Table 1S (Supplementary 1)** displays FLC level indices together with available MRI findings in naive patients: Gd+ lesions were present in the brain and/or spinal cord of each of 10 relapse cases with abnormally high FLC levels. In contrast, in 2 of 3 relapse cases showing normal FLC levels no Gd+ lesions were present. MRI data were available in 9 of 10 remission cases; in 8 of them, no Gd+ lesions were found, and all of these showed FLC levels within the normal range. The analysis of obtained data suggests correlation between saliva FLC levels and MRI findings. This correlation was checked statistically by comparing two logistic regression models, one based on FLC findings (**Supplementary 2**), the second one – on those of MRI (**Supplementary 3**). If both of these two models provide effective discrimination between the disease states, the logit values of these models should correlate.

The discriminating ability of the FLC based on logistic regression (cFLC-F) is described above. The Firth’s logistic regression model for MRI data was constructed using two predictors: the load of T2 in the brain, [(T2)_brain_], and Gd*+* in the brain&spine, [Sum(Gd+)] (**Supplementary 3**).

The corresponding logit, cMRI-F, is defined as


(Eq.2) $$cMRI–F=logit\left[Pr\left(Y=1\right)\right]=-4.18+0.2{\left(T2\right)}_{brain}+2.46\left[Sum\left(Gd+\right)\right]$$

**Figure 4:**
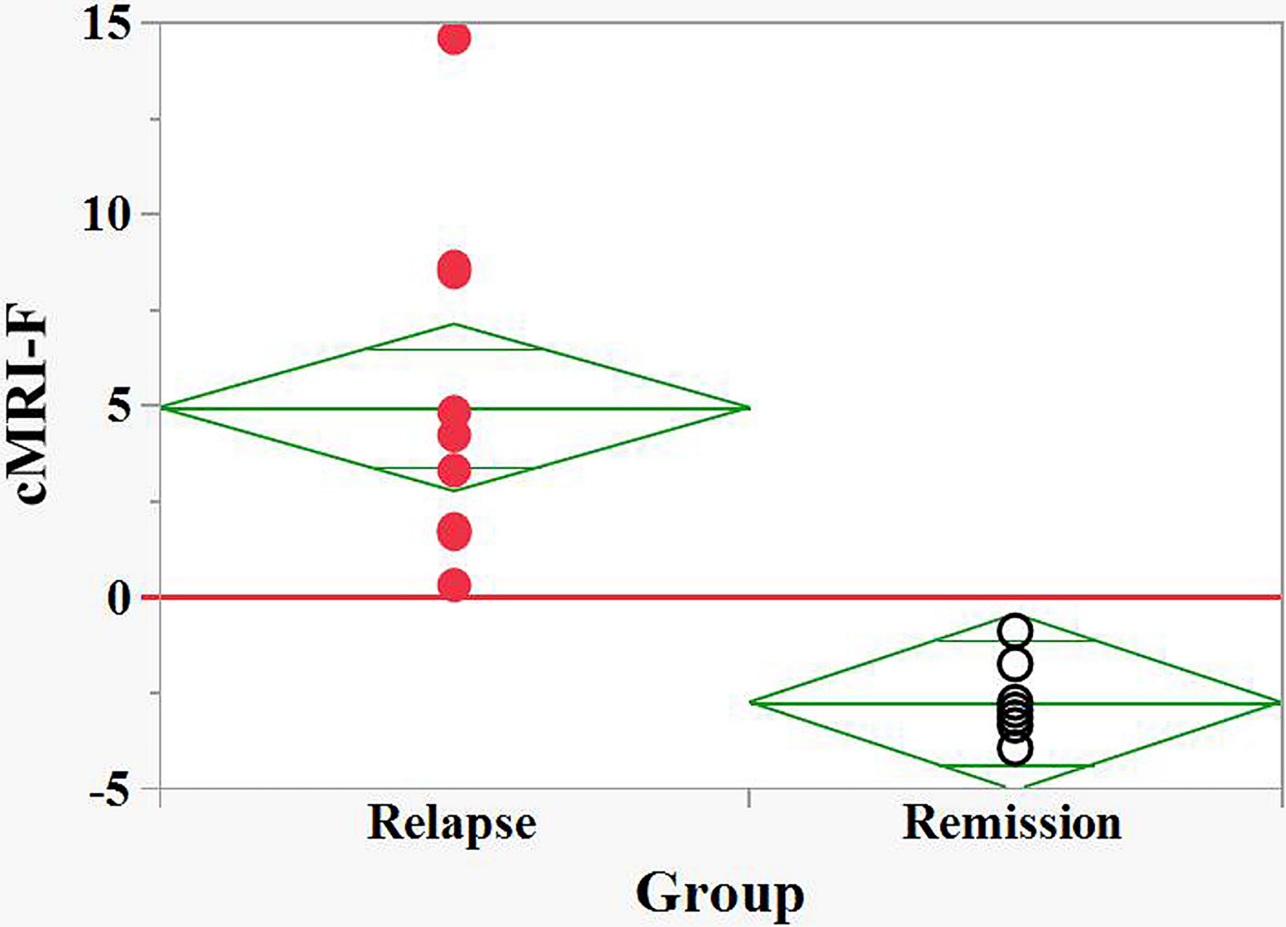
Discrimination between relapse and remision groups of naive POMS patients by the MRI based Firth logistic regression function, cMRI-F. cMRI-F values were calculated for each individual (**Supplement 2**). “Relapse” - red filled circles, “Remission” - black empty circles. Effective discrimination between the POMS patients in relapse and remission groups was observed (cut-off value, cFLC-F = 0).

This MRI model allows effective discrimination between relapse and remission groups (**Figure 4**).

Finally, we demonstrated a non-parametric correlation at significance level α = 0.05 between the cFLC-F and cMRI-F values, with the Spearman’s *ρ* = 0.54 (*p*-value = 0.017) (**Supplementary 4**). *This finding confirms the utility of the saliva FLC analysis for assessing disease activity in naive POMS patients.*

Treatment of POMS patients may reduce the saliva FLC levels, especially for those under CS treatment

### DMT Treatment

Of 3 treated MS relapse cases, the saliva FLC levels were normal in 2 and abnormal in 1 case. Of the 23 patients in remission, the FLC levels were normal in 18, abnormal in 3, and borderline in 2 patients (**Table 1**, **Supplementary 1** and **Table S2**).

### CS Treatment

Four MS patients in relapse/post relapse state were treated with CS and tested during or shortly after (1-5 weeks) their treatment (**Table 1**, **Supplementary 1** and **Table S2**). The FLC levels were normal in 3 of 4 relapse case and abnormally high in one remaining relapse case. Of note, in the latter relapse case (a) the load of Gd+ lesions was markedly high (comparing to other 3 CS treated relapse cases), and this patient experienced additional attacks shortly after finishing the CS treatment (2 and 5 months later).

### FLC Test in POMS Patients Follow-Up

The above presented data show strong link between the values of saliva FLC levels and patient clinical status, radiological findings and treatment. These findings suggest a possible utility of FLC test for the follow-up of individual patients, as it was shown below.

**Figure 5:**
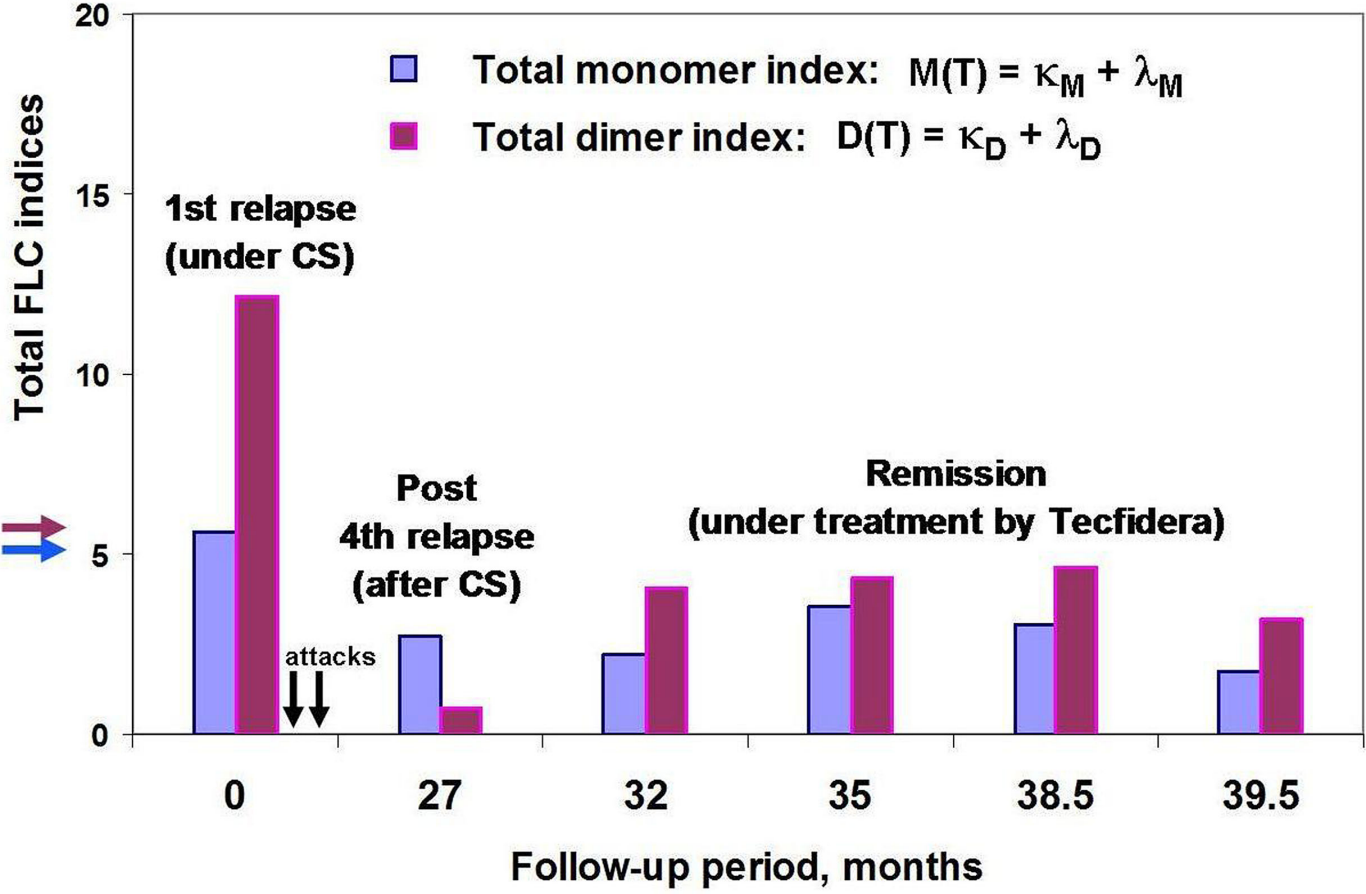
POMS patient #46 follow-up using saliva FLC test. Vertical axis y shows total FLC monomer and dimer indices, namely, M(T) = (κ_M_ + λ_M_) – blue, D(T) = (κ_D_ + λ_D_) – purple. The arrows on the vertical axis **y** (blue and purple) indicate the cut-off values of indices to distinguish between normal and abnormally high FLC levels. Horizontal axis ***x*** – follow-up period (months); the arrows indicate the second and third attacks, 2 and 5 months after the first one (no saliva samples were available). Note that the FLC values at the 27 month of patient follow-up (post-relapse) were obtained by analysis of saliva sample collected on the 20^th^ day after acute attack and on the 6^th^ day after the ending the treatment with oral prednisone.

**Figure 6:**
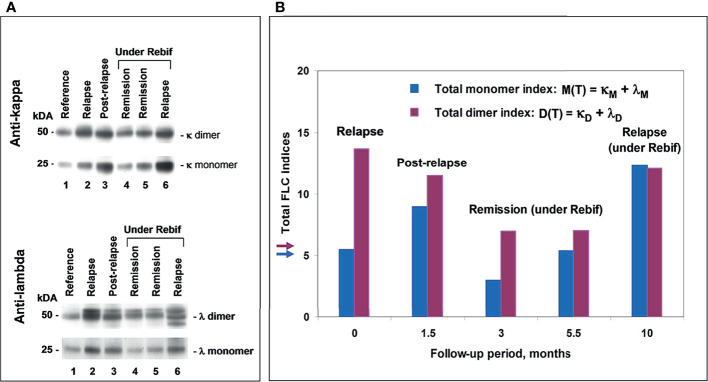
POMS patient #5 follow-up using saliva FLC test. **(A)** Western blotting of κ and λ FLC monomers and dimers: track 1 – reference sample; track 2 - patient in relapse (starting point); track 3 - post-relapse, 1.5 months later; tracks 4 and 5 – remission under Rebif, 3 and 5.5 months later, respectfully; track 6 – relapse, 10 months later, with an appearance of new active lesions (under Rebif, before initiation of CS). **(B)** Chart: vertical axis ***y*** shows total FLC monomer and dimer indices, namely, M(T) = (κ_M_ + λ_ M_) – blue, D(T) = (κ_D_ + λ_D_) – purple; horizontal axis ***x*** – follow-up period (months). Arrows indicate the cut-off values of indices to distinguish between normal and abnormally high FLC levels.

Saliva FLC levels vs clinical status during patient follow-up:

Saliva FLC of 17 POMS patients were analyzed repeatedly (from 2 to 7 times) during their follow-up. In 16 of 17 patients transition from relapse to remission states or vice versa was accompanied by reduction or elevation of saliva FLC levels [M(T) and/or D(T)], respectively (**Figures 5** and **6**).

**Figure 7:**
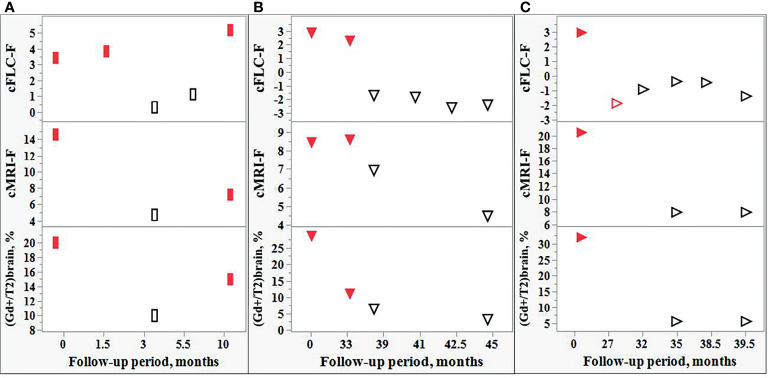
Comparison of the changes in the FLC levels and MRI data during the follow-up of individual POMS patients. Changes in the cFLC-F values are in concordance with those of cMRI-F and also with the relative amount active lesions in the brain, i.e., [(Gd+)/T2]_brain_,%. Red filled rectangles [**(A)**: POMS patient #5] and red filled triangles [**(B, C)**: POMS patients #1 and #46] indicate relapse. Red empty triangle [**(C)**: patient #46] - post-relapse. Black empty rectangles **(A)** and triangles **(B, C)** - remission.

Saliva FLC levels vs MRI data during patient follow-up:

Five of 9 tested patients showed the relapse-remission transitions during their follow-up. The observed changes in the cFLC-F values were usually in concordance with those in (a) cMRI-F, and (b) the relative number of active lesions in the brain, i.e., [(Gd+)/T2]_brain_, % (although the effect of the treatment with CS should be taken into consideration) (**Figure 7**).

Four remaining patients were in clinically stable remission during their follow-up: they showed no changes in their MRI scans and demonstrated normal FLC index values during their follow-up.

### Saliva FLC in the Pediatric Patients With Non-MS Neurological Diseases

Patients with *non-MS demyelinating diseases* included *s/p* CIS (*n* = 17), s/p MOG+ (*n* = 3), s/p ADEM (*n* = 1), as well as patients with RIS (*n* = 3). All these patients showed no Gd+ lesions. The FLC indices were normal in all but 2 CIS cases (**Supplementary 1** and **Table S3**). Another tested subgroup included patients *in relapse* with CIS (*n* = 3), MOG+ (*n* = 1), ADEM (*n* = 1) and NMO/AQP4+ (*n* = 1). Two of 3 CIS patients were treated with CS and showed trace amounts of FLC. The remaining CIS patient in relapse was not treated and showed no Gd+ lesions; his saliva FLC index values were normal. FLC index values were normal in the patient with MOG+ and abnormally high in patients with ADEM and NMO. The MRI scans of the latter 3 cases showed Gd+ lesions (**Supplementary 1** and **Table S3**).

The diagnoses of the 11 tested patients (naive) with non-demyelinating neurological diseases are showed in **Table 2**. The FLC index values were normal in 10 of these 11 patients and borderline in the remaining one.

## Discussion

In view of recent advancements in disease modifying therapies for MS, a range of treatment options are now available for all ranges of severity of the disease. The early initiation or timely switch to a more potent therapy can be justified by using appropriate biomarkers of disease activity. Therefore, the laboratory markers of prognostication and monitoring disease activity have received particular attention. Most of such markers (for example, neurofilament light chains (NfL), glial fibrillary acidic protein (GFAP), YKL-40, immunoglobulin kappa light chains) are found in the CSF (26). However, the invasiveness of the CSF testing diminishes the applicability of these CSF markers for disease monitoring. Despite the potential role of *serum* NfL in MS over the last 5 years, a number of hurdles remain before this test can be integrated into routine clinical practice (27).

In the present study, the technique of the FLC monomer and dimer analysis developed by us previously (21, 22) was applied to study the *saliva* of POMS patients. In *naive* POMS patients the saliva levels of FLC in the relapse group differed significantly from those in remission. As we reported earlier (21), the diurnal fluctuations of FLC monomers and dimers of the same patient were small as compared to the differences in FLC levels observed between relapse and healthy state, as well as between relapse and remission. We found that the abnormally high levels of FLC monomers and/or dimers commonly observed in relapse state, significantly correlated with the load of the Gd+ lesions, while in the absence of active lesions, the saliva FLC levels were usually normal.

In most CS treated cases both pediatric and adult MS patients (22) showed significantly reduced FLC levels in saliva. However, in one POMS patient we observed abnormally high FLC levels after the 1^st^ attack despite CS treatment. Of importance, this patient endured 2 additional attacks shortly after finishing the CS treatment of the 1^st^ attack. In contrast, the FLC levels were low at the 4^th^ (CS-treated) attack which was followed by stable remission (**Figure 5**). Thus, saliva FLC measurements may help in regulating the dosage and duration of CS treatment, as well as in the decisions regarding the second-line treatment.

Some DMT-treated patients have displayed normal FLC values in the presence of a small number of Gd+ lesions (**Supplementary 1** and **Table S2**). However, the follow-up of these patients revealed that the appearance of new active lesions may be accompanied by increased FLC levels and development of a new attack. Together with similar findings in our previous study of the adult MS population (21), these observations indicate a potential value of our FLC test for prognosis and monitoring disease activity (**Figure 6**). Moreover, using the Firth logistic regression analysis we have demonstrated the tendency of FLC level changes to closely follow MRI findings during the follow-up period (**Figure 7**).

The developed saliva FLC test may be of special importance in POMS. At present, MRI is commonly used in the MS patient follow-up (a) to assess the disease dynamics prior to therapeutic decisions, and (b) to evaluate the effect of treatment and activity of the disease. *Keeping in mind the inclination to minimize the exposure of POMS patients to gadolinium, our saliva FLC test could serve as a safe and inexpensive tool for monitoring the disease course*.

A question was raised whether our findings are specific to POMS or are common to a wider spectrum of relapsing-remitting demyelinating diseases. Normal FLC levels were found in 23 of 26 naive patients with CIS, MOG+ and ADEM showing no Gd+ lesions in the brain. However, of 3 patients with Gd+ lesions in the brain, namely, ADEM (*n* = 1), MOG+ (*n* = 1), and NMO/AQP4+ (*n* = 1), two (ADEM and NMO/AQP4+) showed abnormal FLC levels. Thus, despite a low number of the available Gd+ cases, *it is possible that the saliva FLC analysis could be used to assess the disease activity in a wider spectrum of demyelinating diseases*. More pediatric patients should be tested to reach definite conclusions.

Finally, our findings support the view of involvement of mucosal immunity in the pathophysiology of demyelinating diseases. However, the mechanisms leading to the pathological changes in the saliva FLC levels in POMS are not clear and require further investigation. Are the increased FLC levels related to the changes of local synthesis and secretion of immunoglobulins or they are caused by the interplay between local and systemic immunity. However, there is still no consensus regarding this question. While some studies raise possibility of impaired mucosal barriers in MS (28–30), other reports support a strong partitioning of oral from systemic humoral immunity (31). Irrespectively of what are the precise mechanisms explaining the saliva FLC level changes in POMS, our study demonstrates the utility of the non-invasive and safe saliva FLC test for monitoring the disease activity in POMS, and, potentially, in other demyelinating diseases.

## Data Availability Statement

The original contributions presented in the study are included in the article/**Supplementary Material**. Further inquiries can be directed to the corresponding author.

## Ethics Statement

The studies involving human participants were reviewed and approved by Beilinson Medical Center Institutional Review Board (No. 0104-15-RMC). Written informed consent to participate in this study was provided by the participants’ legal guardian/next of kin.

## Author Contributions

EG-C. designed the study, participated in the experimental part of the study, provided clinical information, participated in discussions and interpretation of the obtained results, and in preparation of manuscript. BK designed the study, performed the experiments, participated in discussions and interpretation of the obtained results, and in preparation of manuscript. ET and EK performed the statistical part of the study and participated in preparation of the manuscript. SG participated in the experimental part of the study and discussions. AR participated in the discussions and data acquisition. All authors contributed to the article and approved the submitted version.

## Conflict of Interest

Author ET is employed by Tartakovsky MLD Consultancy, Rishon Lezion, Israel and author EK is employed by Quant Aspect Limited, London, United Kingdom.

The remaining authors declare that the research was conducted in the absence of any commercial or financial relationships that could be construed as a potential conflict of interest.

The handling editor DF declared a shared affiliation with the author EGC at the time of review.

## Publisher’s Note

All claims expressed in this article are solely those of the authors and do not necessarily represent those of their affiliated organizations, or those of the publisher, the editors and the reviewers. Any product that may be evaluated in this article, or claim that may be made by its manufacturer, is not guaranteed or endorsed by the publisher.

## References

[1] Dobson R Giovannoni G. Multiple Sclerosis – A Review. Eur J Neurol (2019) 26(1):27–40. doi: 10.1111/ene.13819 30300457

[2] Ghezzi A Deplano V Faroni J Grasso MJ Liquori M Marrosu G. Multiple Sclerosis in Childhood: Clinical Features of 149 Cases. Mult Scler (1997) 3:43–6. doi: 10.1177/135245859700300105 9160345

[3] Boiko A Vorobeychik G Paty D Devonshire V Sadovnick D. Early Onset Multiple Sclerosis: A Longitudinal Study. Neurology (2002) 59(7):1006–10. doi: 10.1212/wnl.59.7.1006 12370453

[4] Ferreira ML Machado MI Dantas MJ Moreira AJ Souza AM. Pediatric Multiple Sclerosis: Analysis of Clinical and Epidemiological Aspects According to National MS Society Consensus 2007. Arq Neuropsiquiatr (2008) 66:665–70. doi: 10.1590/S0004-282X2008000500011 18949259

[5] Gadoth N. Multiple Sclerosis in Children. Brain Dev (2003) 25(4):229–32. doi: 10.1016/s0387-7604(03)00035-4 12767451

[6] Yan K Balijepalli C Desai K Gullapalli L Druyts E. Epidemiology of Pediatric Multiple Sclerosis: A Systematic Literature Review and Meta-Analysis. Mult Scler Rel Dis (2020) 44(1):102260. doi: 10.1016/j.msard.2020.102260 32540746

[7] Alroughani R Boyko A. Pediatric Multiple Sclerosis: A Review. BMC Neurol (2018) 18(1):27–35. doi: 10.1186/s12883-018-1026-3 29523094 PMC5845207

[8] Polman CH Reingold SC Edan GF Filippi M Hartung H-P Kappos L. Diagnostic Criteria for Multiple Sclerosis: 2005 Revisions to the McDonald Criteria. Ann Neurol (2005) 58(6):840–6. doi: 10.1002/ana.20703 16283615

[9] Polman CH Reingold SC Banwell B Clanet M Cohen JA Filippi M. Diagnostic Criteria for Multiple Sclerosis: 2010 Revisions to the McDonald Criteria. Ann Neurol (2011) 69(2):292–302. doi: 10.1002/ana.22366 21387374 PMC3084507

[10] Thompson AJ Banwell BL Barkhof F Carrol WM Coetzee T Comi G. Diagnosis of Multiple Sclerosis: 2017 Revisions of the Mc Donald Criteria. Lancet Neurol (2018) 17(2):162–73. doi: 10.1016/S1474-4422(17)30470-2 29275977

[11] Dale RC Ramanathan S. Clinical Decision Making in MOG Antibody-Associated Disease. Lancet Neurol (2021) 20(9):695–7. doi: 10.1016/S1474-4422(21)00247-7 34418387

[12] Abdel-Mannan O Cortese R Wassmer E Hemingway C Thompson A Brownlee W. Primary Progressive Multiple Sclerosis Presenting Under the Age of 18 Years: Fact or Fiction? Mult Scler J (2021) 27(2):309–14. doi: 10.1177/1352458520910361 32124676

[13] Ghezzi A Baroncini D Zaffaroni Comi G. Pediatric Versus Adult MS: Similar or Different? Multiple Sclerosis Demyelinating Disord (2017) 2(5):1–14. doi: 10.1186/s40893-017-0022-6

[14] Ghezzi A. Clinical Characteristics of Multiple Sclerosis With Early Onset. Neurol Sci (2004) 25(Suppl 4):S336–9. doi: 10.1007/s10072-004-0336-y 15727228

[15] Huppke B Ellenberger D Rosewich H Friede T Gärtner J Huppke P. Clinical Presentation of Pediatric Multiple Sclerosis Before Puberty. Eur J Neurol (2014) 21(3):441–6. doi: 10.1111/ene.12327 24330201

[16] Traboulsee A Simon JH Stone L Fisher E Jones DE Malhotra A. Revised Recommendations of the Consortium of MS Centers Task Force for a Standardized MRI Protocol and Clinical Guidelines for the Diagnosis and Follow-Up of Multiple Sclerosis. Am J Neuroradiol (2016) 37(3):394–401. doi: 10.3174/ajnr.A4539 26564433 PMC5094650

[17] Hemond CC Bakshi R. Magnetic Resonance Imaging in Multiple Sclerosis. Cold Spring Harb Perspect Med (2018) 8(5):a028969. doi: 10.1101/cshperspect.a028969 29358319 PMC5932576

[18] Kanda T Fukusato T Matsuda M Toyoda K Oba H Kotoku J. Gadolinium-Based Contrast Agent Accumulates in the Brain Even in Subjects Without Severe Renal Dysfunction: Evaluation of Autopsy Brain Specimens With Inductively Coupled Plasma Mass Spectroscopy. Radiology (2015) 276(1):228–32. doi: 10.1148/radiol.2015142690 25942417

[19] Kanda T Ishii K Kawaguchi H Kitajima K Takenaka D. High Signal Intensity in the Dentate Nucleus and Globus Pallidus on Unenhanced T1-Weighted MR Images: Relationship With Increasing Cumulative Dose of a Gadolinium-Based Contrast Material. Radiology (2014) 270(3):834–41. doi: 10.1148/radiol.13131669 24475844

[20] Kaplan B Ganelin-Cohen E Golderman S Livneh A. Diagnostic Utility of Kappa Free Light Chains in Multiple Sclerosis. Exp Rev Mol Diagnost (2019) 19(4):277–9. doi: 10.1080/14737159.2019.1586535 30795689

[21] Kaplan B Golderman S Ganelin-Cohen E Miniovitch A Korf E Ben-Zvi I. Immunoglobulin Free Light Chains in Saliva: A Potential Marker for Disease Activity in Multiple Sclerosis. Clin Exp Immunol (2018) 192(1):7–17. doi: 10.1111/cei.13086 29194592 PMC5842412

[22] Lotan I Ganelin-Cohen E Tartakovsky E Khasminsky V Hellmann M Steiner I. Saliva Immunoglobulin Free Light Chain Analysis for Monitoring Disease Activity and Response to Treatment in Multiple Sclerosis. Mult Scler Relat Disord (2020) 44:102339. doi: 10.1016/j.msard.2020.102339 32599469

[23] Kaplan B Aizenbud BM Golderman S Yaskariev R Sela BA. Free Light Chain Monomers in the Diagnosis of Multiple Sclerosis. J Neuroimmunol (2010) 229(1-2):263–73. doi: 10.1016/j.jneuroim.2010.09.002 20870296

[24] Filippi M Preziosa P Banwell BL Barkhof F Ciccarelli O De Stefano N. Assessment of Lesions on Magnetic Resonance Imaging in Multiple Sclerosis: Practical Guidelines. Brain (2019) 142(7):1858–75. doi: 10.1093/brain/awz144 PMC6598631 31209474

[25] Firth D. Bias Reduction of Maximum Likelihood Estimates. Biometrika (1993) 80:27–38. doi: 10.2307/2336755

[26] Magliozzi R Cross AH. Can CSF Biomarkers Predict Future MS Disease Activity and Severity? Mult Scler J (2020) 26(5):582–90. doi: 10.1177/1352458519871818 31965889

[27] Thebault S Booth RA Rush CA MacLean H Freedman MS. Serum Neurofilament Light Chain Measurement in MS: Hurdles to Clinical Translation. Front Neurosci (2021) 15:1–8. doi: 10.3389/fnins.2021.654942 PMC8027110 33841093

[28] Coyle PK Bulbank M. Immune-Reactive Cells in Multiple Sclerosis Mucosal Secretions. Neurology (1989) 39:378–80. doi: 10.1212/WNL.39.3.378 2927645

[29] Coyle PK. Molecular Analysis of IgA in Multiple Sclerosis. J Neuroimmunol (1989) 22:83–92. doi: 10.1016/0165-5728(89)90038-6 2925844

[30] Nouri M Bredberg A Westr€om B Lavasani S Lees JR. Intestinal Barrier Dysfunction Develops at the Onset of Experimental Autoimmune Encephalomyelitis, and can be Induced by Adoptive Transfer of Auto-Reactive T Cells. PloS One (2014) 9:e106335. doi: 10.1371/journal.pone.0106335 25184418 PMC4153638

[31] Heaney JL Sian Faustini S Evans L Rapson A Collman E Emery A. Investigating the Utility of Saliva Immunoglobulins for the Detection of Myeloma and Using Myeloma Proteins to Clarify Partition Between Oral and Systemic Immunity. Eur J Haematol (2022) 00:1–10. doi: 10.1111/ejh.13758 PMC9314979 35184331

