# Supplementary material for: Personalized Disease Monitoring in Pediatric Onset Multiple Sclerosis Using the Saliva Free Light Chain Test
Source: Front Immunol. 2022 Apr 5;13:821499. doi: 10.3389/fimmu.2022.821499 (PMC9016751; doi:10.3389/fimmu.2022.821499)
Supplement: Supplementary file 1 [file Table_1.doc]

**Supplement 1: Table S1. Naive POMS patients: saliva FLC indices and clinical and radiological findings.**

| **Sample code*** | **FLC indices** | | | | | | **MRI: T2 load** | | | **MRI: Gd+ lesions** | | **Optic nerve enh.**** | **EDSS** |
| --- | --- | --- | --- | --- | --- | --- | --- | --- | --- | --- | --- | --- | --- |
|  | **κ_D_** | **κ_M_** | **λ_D_** | **λ_M_** | **κ_D_+λ_D_** | **κ_M_+λ_M_** | **brain** | | **spine** | **brain** | **spine** |  |  |
| **Non-treated MS patients in relapse** | | | | | | | | | | | | | |
| 1/36 | 2.43 | 9.24 | 4.50 | 1.34 | 6.93 | 10.58 | 14 | | 0 | 4 | 0 | yes | 1 |
| 1/91 | 3.46 | 5.36 | 5.07 | 1.89 | 8.53 | 7.25 | 27 | | 0 | 3 | 0 | no | 2 |
| 2/31 | 1.67 | 0.69 | 2.73 | 21.44 | 4.40 | 22.13 | 17 | | 0 | 1 | 0 | no | 0 |
| 3/37 | 2.53 | 5.78 | 2.91 | 1.34 | 5.44 | 7.12 | 8 | | 3 | 1 | 2 | no | 2 |
| 4/76 | 7.96 | 3.08 | 11.70 | 3.83 | 19.66 | 6.91 | 10 | | 1 | 0 | 1 | no | 1 |
| 5/104 | 3.76 | 3.17 | 9.93 | 2.29 | 13.69 | 5.46 | 20 | | 6 | 4 | 2 | no | 1 |
| 5/110 | 4.20 | 6.64 | 7.25 | 2.29 | 11.45 | 8.93 | n/a*** | | n/a | n/a | n/a | no | 1 |
| 6/148 | 4.26 | 4.88 | 5.20 | 5.48 | 9.46 | 10.36 | 5 | | 0 | 2 | 0 | no | 2 |
| 6/165 | 3.82 | 7.90 | 4.73 | 2.68 | 8.55 | 10.58 | n/a | | n/a | n/a | n/a | no | 1 |
| 7/78 | 2.97 | 7.44 | 4.37 | 2.68 | 7.33 | 10.12 | 25 | | 2 | 1 | 0 | no | 1 |
| 7/86 | 2.81 | 8.73 | 4.37 | 2.52 | 7.17 | 11.25 | n/a | | n/a | n/a | n/a | no | 1 |
| 10/144 | 1.65 | 1.26 | 2.52 | 0.76 | 4.17 | 2.03 | 29 | | 6 | 0 | 0 | no | 2 |
| 11/107 | 2.11 | 2.94 | 11.62 | 4.56 | 13.73 | 7.50 | 2 | | n/a | 0 | n/a | yes | 2 |
| 32/169 | 0.64 | 0.51 | 0.42 | 0.63 | 1.05 | 1.14 | 5 | | 0 | 3 | 0 | no | 1 |
| **Non-treated MS patients in remission** | | | | | | | | | | | | | |
| 6/166 | 2.49 | 3.94 | 2.13 | 0.58 | 4.62 | 4.52 | 4 | 0 | | 0 | 0 | no | 0 |
| 13/87 | 0.90 | 2.34 | 0.44 | 1.34 | 1.34 | 3.68 | 6 | 2 | | 0 | 0 | no | 0 |
| 13/117 | 2.25 | 2.09 | 2.03 | 1.34 | 4.28 | 3.43 | 7 | 2 | | 0 | 0 | no | 0 |
| 14/72 | 0.06 | 0.94 | 0.42 | 1.38 | 0.48 | 2.32 | 5 | 0 | | 0 | 0 | no | 0 |
| 14/120 | 0.02 | 0.89 | 0.05 | 1.34 | 0.07 | 2.23 | 7 | 3 | | 0 | 0 | no | 0 |
| 14/121 | 0.02 | 0.43 | 0.05 | 1.34 | 0.07 | 1.77 | 12 | 3 | | 0 | 0 | no | 0 |
| 16/47 | 0.58 | 0.57 | 0.39 | 0.67 | 0.97 | 1.24 | 1 | 0 | | 0 | 0 | no | 0 |
| 19/32 | 0.74 | 0.39 | 0.10 | 0.00 | 0.84 | 0.39 | 5 | 0 | | 0 | 0 | no | 0 |
| 19/39 | 0.18 | 0.14 | 0.47 | 0.98 | 0.65 | 1.12 | 4 | 0 | | 1 | 0 | no | 0 |
| 11/77 | 2.15 | 2.94 | 2.73 | 1.47 | 4.88 | 4.42 | n/a | n/a | | n/a | n/a | yes | 0 |

* patient number/sample number; **enh. - enhancement; *** n/a - not available.

**Supplement 1: Table S2. POMS patients under treatment: saliva FLC indices and clinical & radiological findings**

| **Sample code*** | **FLC indices** | | | | | | **Treatment** | **MRI: T2 load** | | **MRI: Gd+ lesions** | | **Optic nerve enh.**** | **EDSS** |
| --- | --- | --- | --- | --- | --- | --- | --- | --- | --- | --- | --- | --- | --- |
|  | **κ_D_** | **κ_M_** | **λ_D_** | **λ_M_** | **κ_D_+λ_D_** | **κ_M_+λ_M_** |  | **brain** | **spine** | **brain** | **spine** |  |  |
| **Treated MS patients in relapse** | | | | | | | | | | | | | |
| 5/156 | 4.42 | 9.63 | 7.64 | 2.68 | 12.06 | 12.31 | Rebif | 20 | 3 | 3new | 0 | no | 2 |
| 19/74 | 0.72 | 3.93 | 0.29 | 2.72 | 1.00 | 6.65 | Rebif | 7 | 0 | 0 | 0 | no | 1 |
| 45/109 | 0.08 | 0.00 | 0.08 | 0.51 | 0.16 | 0.51 | Avonex | >10 | 5 | 1 | 0 | no | 1 |
| 18/73 | 0.10 | 0.06 | 0.00 | 0.48 | 0.10 | 0.54 | CS*** | 6 | 2 | 2 | 0 | yes | 1 |
| 47/25 | 1.49 | 1.28 | 1.69 | 1.07 | 3.18 | 2.35 | CS | 2 | 2 | 0 | 0 | yes | 2 |
| 46/67 | 2.87 | 4.02 | 9.28 | 1.59 | 12.15 | 5.61 | CS | 25 | 6 | 8 | 0 | yes | 1 |
| 46/96 | 0.60 | 2.00 | 0.13 | 0.75 | 0.73 | 2.75 | Tecfidera/CS**^#^** | n/a.**** | n/a | n/a | n/a | yes | 1 |
| **Treated MS patients in remission** | | | | | | | | | | | | | |
| 1/119 | 1.19 | 0.51 | 0.96 | 1.34 | 2.16 | 1.85 | Tecfidera | 31 | 0 | 2 | 0 | no | 1 |
| 1/153 | 0.90 | 0.17 | 0.75 | 0.13 | 1.65 | 0.30 | Tecfidera | 40 | 0 | 1 | 0 | no | 1 |
| 2/143 | 1.67 | 1.14 | 1.98 | 1.15 | 3.65 | 2.29 | Tecfidera | 19 | 0 | 0 | 0 | no | 0 |
| 3/83 | 2.55 | 2.49 | 2.60 | 2.51 | 5.15 | 5.00 | Tecfidera | 7 | n/a | 0 | n/a | no | 1 |
| 4/93 | 1.49 | 0.22 | 3.77 | 1.29 | 5.26 | 1.50 | Copaxone | 13 | n/a | 0 | n/a | yes | 1 |
| 5/114 | 2.55 | 1.93 | 4.45 | 1.06 | 6.99 | 2.98 | Rebif | 20 | 3 | 2 | 0 | no | 1 |
| 5/134 | 2.82 | 4.14 | 4.21 | 1.24 | 7.03 | 5.38 | Rebif | n/a | n/a | n/a | n/a | no | 1 |
| 9/85 | 1.00 | 0.89 | 1.12 | 0.00 | 2.11 | 0.89 | Tecfidera | n/a | n/a | n/a | n/a | no | 1 |
| 12/115 | 2.23 | 2.09 | 4.76 | 1.34 | 6.99 | 3.43 | Avonex | 19 | 3 | 0 | 0 | no | 1 |
| 15/71 | 0.72 | 0.00 | 0.29 | 0.40 | 1.00 | 0.40 | Tecfidera | 10 | 0 | 0 | 0 | no | 1 |
| 15/126 | 0.22 | 0.09 | 0.49 | 1.34 | 0.71 | 1.43 | Tecfidera | 13 | 0 | 0 | 0 | no | 1 |
| 18/161 | 2.49 | 1.76 | 2.21 | 1.66 | 4.70 | 3.42 | Rebif | 6 | 3 | 0 | 0 | no | 1 |
| 19/122 | 1.05 | 2.90 | 1.20 | 1.34 | 2.25 | 4.24 | Tecfidera | n/a | n/a | n/a | n/a | no | 1 |
| 36/68 | 0.88 | 0.00 | 1.82 | 0.00 | 2.70 | 0.00 | Rebif | 18 | 0 | 0 | 0 | no | 1 |
| 45/111 | 1.09 | 0.05 | 1.14 | 1.34 | 2.24 | 1.39 | Avonex | 12 | 5 | 0 | 0 | yes | 2 |
| 45/163 | 0.54 | 0.80 | 1.43 | 0.58 | 1.97 | 1.38 | Avonex | 12 | 5 | 0 | 0 | yes | 1 |
| 45/164 | 1.15 | 1.48 | 2.00 | 1.10 | 3.16 | 2.58 | Avonex | 12 | 5 | 0 | 0 | yes | 1 |
| 46/118 | 1.65 | 0.89 | 2.42 | 1.34 | 4.07 | 2.23 | Tecfidera | n/a | n/a | n/a | n/a | no | 1 |
| 46/154 | 1.87 | 1.82 | 2.47 | 1.74 | 4.34 | 3.56 | Tecfidera | 36 | 3 | 2 | 0 | no | 1 |
| 46/157 | 1.87 | 1.99 | 2.78 | 1.05 | 4.65 | 3.03 | Tecfidera | n/a | n/a | n/a | n/a | no | 1 |
| 46/167 | 1.47 | 1.54 | 1.72 | 0.20 | 3.19 | 1.74 | Mavenclad | 36 | 3 | 2 | 0 | no | 1 |
| 48/162 | 0.80 | 0.92 | 1.35 | 0.82 | 2.15 | 1.74 | Tecfidera | 5 | 0 | 1 | 0 | no | 1 |
| 48/159 | 0.84 | 1.25 | 1.38 | 0.76 | 2.21 | 2.01 | Tecfidera | 5 | 1 | 1 | 0 | no | 2 |

* patient number/sample number; **enh. - enhancement; *** corticosteroids; ****; n/a - not available; ^#^post-relapse.

**Supplement 1:Table S3. Pediatric patients with non MS demyelinating: diagnosis, saliva FLC indices, and clinical and radiological findings**

| **Sample code*** | **FLC indices** | | | | | | **Diagnosis** | **MRI: T2 load** | | **MRI: Gd+ lesions** | | **Optic nerve enh.**** | **EDSS** |
| --- | --- | --- | --- | --- | --- | --- | --- | --- | --- | --- | --- | --- | --- |
|  | **κ_D_** | **κ_M_** | **λ_D_** | **λ_M_** | **κ_D_+λ_D_** | **κ_M_+λ_M_** |  | **brain** | **spine** | **brain** | **spine** |  |  |
| 20/24 | 1.87 | 3.45 | 1.92 | 0.27 | 3.79 | 3.72 | s/p CIS *** | 2 | 0 | 0 | 0 | no | 0 |
| 21/13 | 0.40 | 0.20 | 0.68 | 0.43 | 1.07 | 0.63 | s/p CIS | 2 | 0 | 0 | 0 | no | 0 |
| 22/15 | 0.42 | 0.25 | 0.75 | 0.15 | 1.17 | 0.39 | s/p CIS | 7 | 0 | 0 | 0 | no | 0 |
| 23/141 | 1.00 | 0.25 | 0.26 | 0.15 | 1.26 | 0.39 | s/p CIS | 5 | 0 | 0 | 0 | no | 0 |
| 25/14 | 0.20 | 0.77 | 0.18 | trace | 0.38 | 0.77 | s/p CIS | 1 | 0 | 0 | 0 | no | 0 |
| 26/106 | 1.55 | 0.42 | 1.17 | 0.67 | 2.72 | 1.09 | s/p CIS | 3 | 0 | 0 | 0 | yes | 0 |
| 27/128 | 0.58 | 0.37 | 2.31 | 1.34 | 2.89 | 1.71 | s/p CIS | 2 | 0 | 0 | 0 | yes | 1 |
| 27/130 | 1.75 | 0.18 | 7.72 | 1.34 | 9.47 | 1.52 | s/p CIS | 2 | 0 | 0 | 0 | no | 0 |
| 28/123 | 1.75 | 0.18 | 0.47 | 1.34 | 2.22 | 1.52 | s/p CIS | 0 | 0 | 0 | 0 | yes | 1 |
| 28/131 | 0.12 | 0.18 | 0.18 | 1.34 | 0.30 | 1.52 | s/p CIS | 0 | 0 | 0 | 0 | yes | 0 |
| 29/127 | 0.42 | 0.18 | 0.83 | 1.34 | 1.25 | 1.52 | s/p CIS | 2 | 0 | 0 | 1 | no | 0 |
| 30/95 | 3.48 | 2.25 | 2.94 | 0.58 | 6.42 | 2.82 | s/p CIS | 5 | 0 | 0 | 0 | no | 1 |
| 31/17 | 1.87 | 0.94 | 3.17 | 2.68 | 5.04 | 3.62 | s/p CIS | 2 | 0 | 0 | 0 | no | 0 |
| 32/80 | 0.84 | 0.39 | 0.81 | 0.11 | 1.64 | 0.49 | s/p CIS | 1 | 1 | 0 | 0 | no | 1 |
| 33/146 | 1.07 | 1.49 | 0.73 | 0.95 | 1.80 | 2.45 | CIS acute | 0 | 0 | 0 | 0 | yes | 1 |
| 34/137 | 0.16 | 0.14 | 0.03 | 0.19 | 0.19 | 0.33 | s/p CIS | 4 | 3 | 0 | 0 | no | 1 |
| 35/147 | 0.72 | 1.03 | 0.73 | 0.12 | 1.44 | 1.15 | s/p CIS | 0 | 0 | 0 | 0 | no | 0 |
| 52/155 | 0.66 | 1.09 | 1.14 | 2.14 | 1.80 | 3.24 | s/p CIS | 1 | 0 | 0 | 0 | no | 0 |
| 49/20 | trace | trace | trace | trace | trace | trace | CIS acute | 4 | 1 | 0 | 1 | no | 1 |
| 50/X | trace | trace | trace | trace | trace | trace | CIS acute | 0 | 5 | 0 | 0 | no | 1 |
| 37/81 | 1.65 | 1.43 | 1.59 | 0.00 | 3.24 | 1.43 | s/p MOG episode | 1 | 0 | 0 | 0 | yes | 2 |
| 38/79 | 1.57 | 0.89 | 0.73 | 0.00 | 2.30 | 0.89 | s/p MOG episode | 0 | 0 | 0 | 0 | yes | 0 |
| 39/88 | 1.51 | 4.00 | 1.25 | 1.34 | 2.76 | 5.34 | s/p MOG episode | 0 | 0 | 0 | 0 | yes | 1 |
| 53/170 | 0.28 | 0.28 | 0.13 | 0.08 | 0.41 | 0.36 | MOG –acute | 22 | 8 | 6 | 4 | yes | 1 |
| 40/136 | 1.99 | 1.32 | 2.08 | 1.65 | 4.07 | 2.97 | RIS | 7 | 0 | 0 | 0 | no | 0 |
| 41/12 | 0.92 | 0.32 | 0.00 | 0.11 | 0.92 | 0.43 | RIS | 5 | 0 | 0 | 0 | no | 0 |
| 42/142 | 1.57 | 0.62 | 0.26 | 0.15 | 1.83 | 0.76 | RIS | 5 | 0 | 3 | 0 | no | 0 |
| 43/45 | 3.98 | 15.40 | 5.20 | 13.40 | 9.18 | 28.80 | ADEM acute | 7 | 3 | 3 | 0 | no | 5 |
| 53/168 | 0.38 | 0.75 | 0.73 | 1.34 | 1.11 | 2.09 | s/p ADEM episode | 1 | 0 | 0 | 0 | no | 0 |
| 51/23 | 3.10 | 8.56 | 6.11 | 11.26 | 9.21 | 19.82 | NMO/AQP4+ acute | 0 | 0 | 6 | 3 | yes | 2 |

* patient number/sample number; **enh. - enhancement.
