# Supplementary material for: Personalized Disease Monitoring in Pediatric Onset Multiple Sclerosis Using the Saliva Free Light Chain Test
Source: Front Immunol. 2022 Apr 5;13:821499. doi: 10.3389/fimmu.2022.821499 (PMC9016751; doi:10.3389/fimmu.2022.821499)
Supplement: Supplementary file 2 [file Table_2.doc]

**Supplemental material 2.**

The ability of the saliva FLC test to discriminate between relapse and remission states was studied using logistic regression model

where *β_0_* and *β_j_* are the coefficients of the model, estimated by Firth method designed to control sample bias, particularly in small samples.

Pr(*Y* = 1) = probability of an individual to belong to the relapse group.

This model made it possible to achieve effective discrimination between the relapse and remission states in POMS by using [D(T) + M(T)] as a single variable/predictor ***X***:


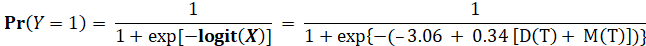


The logit of this model was defined as a **combined FLC index**, the **cFLC-F;** the coefficients of this model are equal to - 3.06 and 0.34 for the intercept (*β*_0_) and the slope (*β*_1_), respectively:

**cFLC-F = logit***(****X****) =* **logit** [Pr(*Y* = 1)] = *β*_0_ + *β_1_X_1_* = –3.06 + 0.34 [D(T) + M(T)]

*X_1_ –* the combination of the FLC indices providing the best discrimination between the groups,

The Supplemental Table S1 presents the cFLC-F index values and the corresponding probabilities for an individual to be in the relapse state according to the model. The cut-off value of probability Pr ≥ 0.5 was used to assign the individuals to the relapse state.

**Supplemental Table S4.** Determination of MS state using logistic regression model based on the cFLC-F

| Sample code | Group | Age | Gender | Tot(D+M) | cFLC-F | Pr[Relapse] | MS state by cFLC-F |
| --- | --- | --- | --- | --- | --- | --- | --- |
| 1/36 | Relapse | 14.5 | 1 | 17.51 | 2.87 | 0.95 | Relapse |
| 1/91 | Relapse | 17 | 1 | 15.78 | 2.29 | 0.91 | Relapse |
| 2/31 | Relapse | 10 | 0 | 26.53 | 5.92 | 1.00 | Relapse |
| 3/37 | Relapse | 17 | 1 | 12.56 | 1.20 | 0.77 | Relapse |
| 4/76 | Relapse | 17 | 1 | 26.57 | 5.94 | 1.00 | Relapse |
| 5/104 | Relapse | 17 | 1 | 19.15 | 3.43 | 0.97 | Relapse |
| 5/110 | Relapse | 17.5 | 1 | 20.38 | 3.84 | 0.98 | Relapse |
| 6/148 | Relapse | 12.5 | 1 | 19.82 | 3.65 | 0.97 | Relapse |
| 6/165 | Relapse | 13 | 1 | 19.13 | 3.42 | 0.97 | Relapse |
| 7/78 | Relapse | 13 | 1 | 17.46 | 2.85 | 0.95 | Relapse |
| 7/86 | Relapse | 13.5 | 1 | 18.43 | 3.18 | 0.96 | Relapse |
| 10/144 | Relapse | 15.5 | 1 | 6.19 | -0.96 | 0.28 | Non-relapse |
| 11/107 | Relapse | 11 | 0 | 21.23 | 4.13 | 0.98 | Relapse |
| 32/169 | Relapse | 16 | 0 | 2.2 | -2.31 | 0.09 | Non-relapse |
| 6/166 | Remission | 13.5 | 1 | 9.14 | 0.04 | 0.51 | Relapse |
| 13/87 | Remission | 17 | 1 | 5.02 | -1.36 | 0.21 | Non-relapse |
| 13/117 | Remission | 17 | 1 | 7.71 | -0.44 | 0.39 | Non-relapse |
| 14/72 | Remission | 16 | 0 | 2.8 | -2.11 | 0.11 | Non-relapse |
| 14/120 | Remission | 16.5 | 0 | 2.3 | -2.28 | 0.09 | Non-relapse |
| 14/121 | Remission | 17.5 | 0 | 1.84 | -2.43 | 0.08 | Non-relapse |
| 16/47 | Remission | 14 | 1 | 2.21 | -2.31 | 0.09 | Non-relapse |
| 19/32 | Remission | 14.5 | 1 | 1.23 | -2.64 | 0.07 | Non-relapse |
| 19/39 | Remission | 15 | 1 | 1.77 | -2.45 | 0.08 | Non-relapse |
| 11/77 | Remission | 10 | 0 | 9.29 | 0.09 | 0.52 | Relapse |
| CH1 | Healthy | 14 | 1 | 3.71 | -1.80 | 0.14 | Non-relapse |
| CH4 | Healthy | 10 | 0 | 4.47 | -1.54 | 0.18 | Non-relapse |
| CH5 | Healthy | 14 | 1 | 6.25 | -0.94 | 0.28 | Non-relapse |
| CH6 | Healthy | 17 | 1 | 6.5 | -0.85 | 0.30 | Non-relapse |
| CH8 | Healthy | 16 | 1 | 2.4 | -2.24 | 0.10 | Non-relapse |
| CH11 | Healthy | 15 | 0 | 1.99 | -2.38 | 0.08 | Non-relapse |
| CH7 | Healthy | 17 | 1 | 3.3 | -1.94 | 0.13 | Non-relapse |
| CH2 | Healthy | 14 | 1 | 3.35 | -1.92 | 0.13 | Non-relapse |
| CH14 | Healthy | 17 | 1 | 2.01 | -2.37 | 0.09 | Non-relapse |
| CH15 | Healthy | 15 | 0 | 0.74 | -2.80 | 0.06 | Non-relapse |
| CH9 | Healthy | 16 | 1 | 2.53 | -2.20 | 0.10 | Non-relapse |
| CH12 | Healthy | 17 | 1 | 6.75 | -0.77 | 0.32 | Non-relapse |
| CH10 | Healthy | 14 | 1 | 5.01 | -1.36 | 0.20 | Non-relapse |
| CH3 | Healthy | 10 | 0 | 2.19 | -2.31 | 0.09 | Non-relapse |
