# Supplementary material for: Personalized Disease Monitoring in Pediatric Onset Multiple Sclerosis Using the Saliva Free Light Chain Test
Source: Front Immunol. 2022 Apr 5;13:821499. doi: 10.3389/fimmu.2022.821499 (PMC9016751; doi:10.3389/fimmu.2022.821499)
Supplement: Supplementary file 3 [file Table_3.doc]

**Supplemental material 3.**

The logistic regression modeling was performed to construct the combined MRI index (cMRI-F) allowing the best discrimination between the relapse and remission states in MS.

The ability of MRI tests to discriminate between relapse and remission states was studied using logistic regression model

where *β_0_* and *β_j_* are the coefficients of the model, estimated by Firth method designed to control sample bias, particularly in small samples.

Pr(*Y* = 1) = probability of an individual to belong to the relapse group.

The logit form of the model obtained is

**logit***(****X****) =* **logit** [Pr(*Y* = 1)] = *β*_0_ + Σ*β_j_X_j_* = – 4.18 + 0.2(T2)_b_ + 2.46[Sum(Gd+)] = **cMRI-F,**

where

*Y* = 1 for the relapse group, *Y* = 0 for the control or remission group;

*X _j_ –*  MRI indices providing the best discrimination between the groups;

*β_0_* = -4.18, *β_1_* = 0.2, and *β_2_* = 2.46 are the coefficients of the model, estimated by Firth method;

(T2)_b_ – the load of T2 lesions in the brain;

Sum(Gd+) – total load of the enhanced lesions in the brain and spine.

**The Supplemental Table S5** presents the cMRI-F index values and the corresponding probabilities for individuals to be in the relapse state according to the model. The cut-off value of probability Pr ≥ 0.5 was used to assign the individuals to the relapse state.

**Supplemental Table S5.** Determination of the MS state using logistic regression model based on **cMRI-F**

| Sample code | Group | Age | Gender | T2b | Sum(Gd+) | (Gd+/T2)b, % | cMRI-F | Pr(Relapse) | MS state  by cMRI-F |
| --- | --- | --- | --- | --- | --- | --- | --- | --- | --- |
| 1/36 | Relapse | 14.5 | 1 | 14 | 4 | 28.57 | 8.46 | 1.00 | Relapse |
| 1/91 | Relapse | 17 | 1 | 27 | 3 | 11.11 | 8.60 | 1.00 | Relapse |
| 2/31 | Relapse | 10 | 0 | 17 | 1 | 5.88 | 1.68 | 0.84 | Relapse |
| 3/37 | Relapse | 17 | 1 | 8 | 3 | 12.5 | 4.80 | 0.99 | Relapse |
| 4/76 | Relapse | 17 | 1 | 10 | 1 | 0 | 0.28 | 0.57 | Relapse |
| 5/104 | Relapse | 17 | 1 | 20 | 6 | 20 | 14.58 | 1.00 | Relapse |
| 6/148 | Relapse | 12.5 | 1 | 5 | 2 | 40 | 1.74 | 0.85 | Relapse |
| 7/78 | Relapse | 13 | 1 | 25 | 1 | 4 | 3.28 | 0.96 | Relapse |
| 10/144 | Relapse | 15.5 | 1 | 29 | 0 | 0 | 1.62 | 0.83 | Relapse |
| 32/169 | Relapse | 16 | 0 | 5 | 3 | 60 | 4.20 | 0.99 | Relapse |
| 6/166 | Remission | 13.5 | 1 | 4 | 0 | 0 | -3.38 | 0.03 | Non-relapse |
| 13/87 | Remission | 17 | 1 | 6 | 0 | 0 | -2.98 | 0.05 | Non-relapse |
| 13/117 | Remission | 17 | 1 | 7 | 0 | 0 | -2.78 | 0.06 | Non-relapse |
| 14/72 | Remission | 16 | 0 | 5 | 0 | 0 | -3.18 | 0.04 | Non-relapse |
| 14/120 | Remission | 16.5 | 0 | 7 | 0 | 0 | -2.78 | 0.06 | Non-relapse |
| 14/121 | Remission | 17.5 | 0 | 12 | 0 | 0 | -1.78 | 0.14 | Non-relapse |
| 16/47 | Remission | 14 | 1 | 1 | 0 | 0 | -3.98 | 0.02 | Non-relapse |
| 19/32 | Remission | 14.5 | 1 | 5 | 0 | 0 | -3.18 | 0.04 | Non-relapse |
| 19/39 | Remission | 15 | 1 | 4 | 1 | 25 | -0.92 | 0.28 | Non-relapse |
