# Supplementary material for: Personalized Disease Monitoring in Pediatric Onset Multiple Sclerosis Using the Saliva Free Light Chain Test
Source: Front Immunol. 2022 Apr 5;13:821499. doi: 10.3389/fimmu.2022.821499 (PMC9016751; doi:10.3389/fimmu.2022.821499)
Supplement: Supplementary file 4 [file Table_4.doc]

**Supplemental material 4.**

Successful follow-up of disease course using saliva FLC test implies that the changes of FLC level reflect the changes in clinical state/MRI findings. If so, we should see statistically significant positive correlation between FLC characteristics on one hand, and MRI/clinical characteristics of patients on another hand. More specifically, if we develop two logistic regression models (one based on FLC characteristics, the second one – on those of MRI), the logits of both models providing discrimination between disease states, should correlate. Our calculations demonstrated the statistically significant correlation at the level α = 0.05 between two logits, cFLC-F and cMRI-F (Supplemental Fig. S1).

**
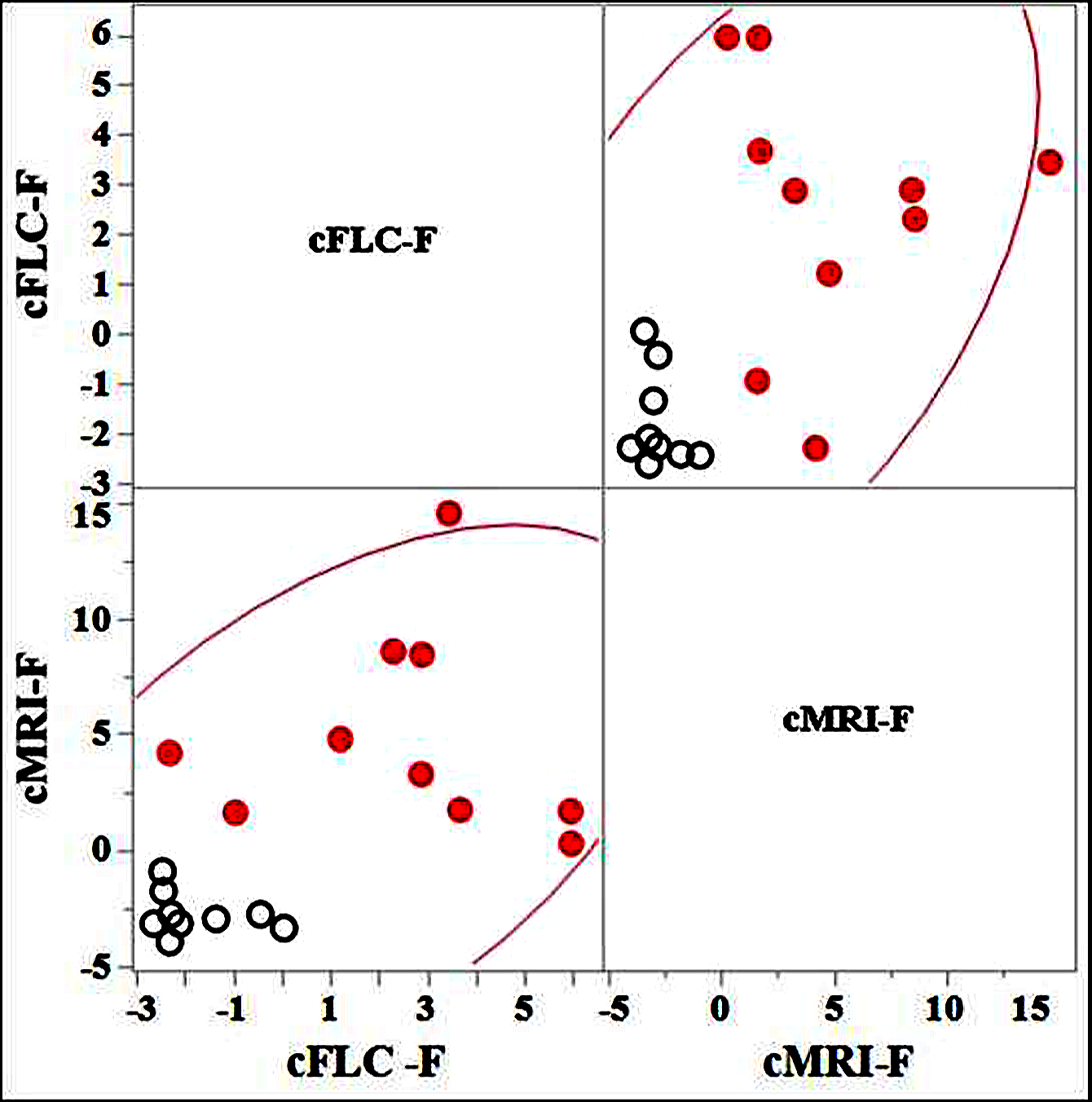
**

**Supplemental Fig. S1**.Correlation between the cFLC-F and cMRI-F values

(Spearman’s ***ρ*** = 0.54; ***p***-value = 0.017) of naive POMS patients. Red filled circles indicate the relapse state, and black empty circles - the remission state.
